# Magnetic bead-based salivary peptidome profiling for periodontal-orthodontic treatment

**DOI:** 10.1186/1477-5956-10-63

**Published:** 2012-11-06

**Authors:** Jieni Zhang, Shaonan Zhou, Ruoxuan Li, Tian Cao, Hui Zheng, Xuedong Wang, Yanheng Zhou, Ning Du, Feng Chen, Jiuxiang Lin

**Affiliations:** 1 Departments of Orthodontics and Central Laboratory, School of Stomatology, Peking University, #22 Zhongguancun South Road, Haidian District, Beijing, 100081, People's Republic of China; 2 Department of Central Laboratory, School of Stomatology, Peking University, Beijing, 100081, People's Republic of China; 3 Department of Stomatology, Beijing An Zhen Hospital, Capital University of Medical Sciences, Beijing, 100081, People's Republic of China

**Keywords:** Periodontal-orthodontic treatment, Peptidome, Saliva, MALDI-TOF MS

## Abstract

**Background:**

Patients with periodontitis seek periodontal-orthodontic treatment to address certain functional and aesthetic problems. However, little is known of the effect of periodontitis on orthodontic treatment. Thus, we compared the differences in peptide mass fingerprints of orthodontic patients with and without periodontitis by MALDI-TOF MS using a magnetic bead-based peptidome analysis of saliva samples. In this way, we aimed to identify and explore a panel of differentially-expressed specific peptides.

**Results:**

Saliva samples from 24 patients (eight orthodontic patients without periodontitis, eight with periodontitis and another eight with periodontitis but no orthodontic treatment) were analyzed, and peptide mass fingerprints were created by scanning MS signals using matrix-assisted laser desorption/ionization time-of-flight mass spectrometry (MALDI-TOF MS) combined with magnetic beads. Nine mass peaks showed significant differences. Orthodontic patients in the group without periodontal disease showed higher mass peaks for seven peptides of the nine, whereas the mass peaks for the other two peptides were higher in the periodontal-orthodontic patients. Besides, these differentially-expressed peptides were sequenced.

**Conclusions:**

The elucidated candidate biomarkers indicated interactions between periodontal condition and orthodontic treatment and their contributions to the changes of saliva protein profiles. Our results provide novel insight into the altered salivary protein profile during periodontal-orthodontic treatment, and may lead to the development of a therapeutic monitoring strategy for periodontics and orthodontics.

## Background

Periodontal-orthodontic treatment is usually needed to correct malocclusions, and may also improve periodontal health [1]. Patients who have chronic periodontal problems want to improve not only their malocclusions to obtain a perfect facial and tooth profile with orthodontic treatment, but also their periodontal condition. However, some doctors believe that orthodontic treatment aggravates periodontal damage [2]; the matter remains disputed. The difference in osteogenesis between periodontitis and non-periodontitis patients [3] may lead to differences in the effect of orthodontic treatment [4]. The aim of this study was to determine the differences between periodontal and non-periodontal orthodontic patients.

Bacterial plaque is an etiologic factor in periodontitis [5]. The pathogenesis of this disease in adults results in a loss of connective tissue, bone support, and, ultimately, teeth [6]. The diagnosis of periodontitis and identification of at-risk patients are challenging. Periodontal condition is usually determined visually by clinical periodontal and X-ray examinations, which are subject to considerable measurement error due to clinical experience, and are often poorly tolerated by patients.

Saliva is a complex hypotonic hydrated solution [7] that contains 3397 different proteins with a variety of biological functions [8]. Whole saliva comprises the secretions of the major and minor saliva glands, non-salivary gingival crevicular fluid, bronchial secretions, bacteria and bacterial products, deciduous epithelial cells, and food debris. Moreover, saliva contains molecules which could be diffused or filtrated from blood [9]. For example, human C-reactive protein (CRP), which is used to assess the coronary events, could be present in saliva [10]. Periodontitis can involve the modified production or release of various factors, such as hormones and cytokines. We sought to discover a candidate biomarker that can facilitate the detection of periodontal changes before pathological symptoms occur.

Since saliva contains multiple potentially informative components and its collection is non-invasive, low-cost, and simple, research is increasingly focusing on the analysis of oral and systemic conditions using saliva as a detection strategy [11,12]. Moreover, there are databases ( http://bioinformatics.ua.pt/OralCard/ and http://www.hspp.ucla.edu/) that compile proteomics data from oral cavity, contributing to the salivary analysis. And protein profiling methods such as two-dimensional polyacrylamide gel electrophoresis for separation and MS for identification have been used to investigate various conditions and disorders, including breast cancer [13], Sjögren’s syndrome [14], rheumatoid arthritis [15], and oral pathologies such as oral cancer [16,17], dental caries [18], cleft palate [19], and periodontitis [20].

We used matrix-assisted laser desorption/ionization time-of-flight mass spectrometry (MALDI-TOF MS), a sensitive MS-based proteomic technique, to detect peptides over a large mass range. The mass spectra generated by this technique are easy to interpret [21]. Here, MALDI-TOF MS was used in combination with WCX to select peptides in the range of 1000–10000 Da prior to further identification. The effectiveness of this combination of techniques has been confirmed in many serum-based peptide profile identification studies [22,23]. MALDI-TOF MS generated accurate salivary protein profiles of patients with fixed orthodontic appliances with and without periodontitis.

In this study, the peptide mass profiles of saliva samples were investigated using magnetic bead-based peptidome analysis. In this way, we aimed to identify a panel of differentially-expressed specific candidate biomarkers.

## Results

**Figure 1:**
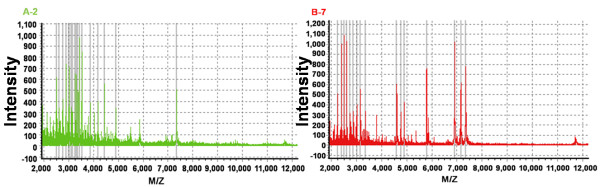
**Complete mass spectra graph.** Complete mass spectra in the range 1000–9000 Da, showing the peptide fingerprints of a saliva sample from a single patient in each group: orthodontic patients without periodontitis (**A**); orthodontic patients with periodontitis (**B**). *m/z*, mass-to-charge ratio.

To investigate the differences between periodontal-orthodontic and non-periodontal orthodontic patients, the entire mass spectra of the extracted peptide samples from 24 subjects (eight per group) were obtained by MALDI-TOF MS (Figure 1). Saliva peptidome fingerprint peaks were characterized in each patient by showing the maximum intensity within a particular *m/z* range. The molecular weight of the majority of the peptides was 1000–7000 Da. The mass spectra peaks were then quantified and compared.

**Figure 2:**
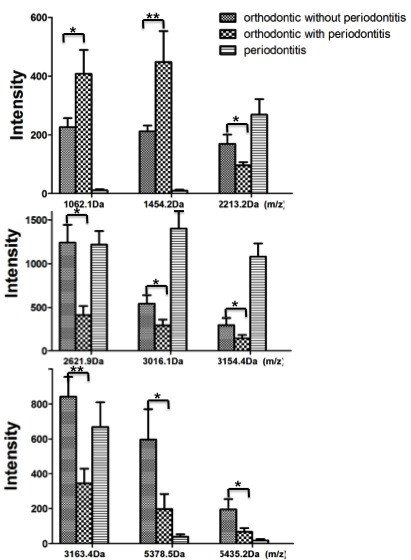
**Column view of the mass spectra of the three groups.** The peak intensities of the three different groups showed differential expression of nine salivary peptides. Noticeably, significant differences were investigated between the two orthodontic groups. (*p <0.05; **p <0.01).

**Figure 3:**
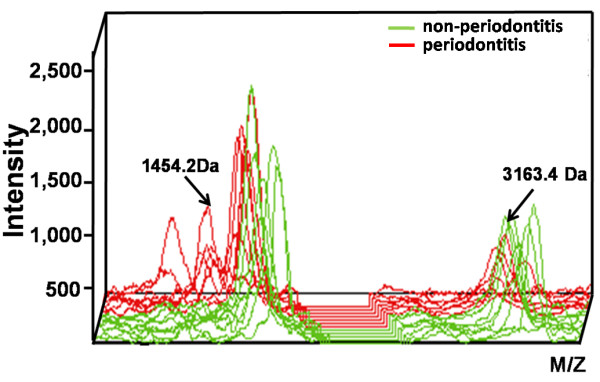
**Three-dimensional *m/z *ratio-intensity maps showing significantly different proteins.** Peptides of 1454.2-Da and 3163.4-Da were the two that were most significantly different. Green curve, non-periodontal orthodontic group; red curve, periodontal-orthodontic group.

An average of 109 protein mass peaks was found. The peak intensities of nine (1062.1, 1454.2, 2213.2, 2621.9, 3016.1, 3154.4, 3163.4, 5378.5, and 5435.2 Da) differed significantly among the three different groups (Table 1). Orthodontic patients in the group without periodontal disease showed higher mass peaks for peptides of 2213.2, 2621.9, 3016.1, 3154.4, 3163.4, 5378.5, and 5435.2 Da, whereas the mass peaks for the peptides of 1062.1 and 1454.2 Da were higher in the periodontal-orthodontic patients (Figures 2 and 3).

**Table 1:** **Significant (*p *<0.05) *m/z *values discriminating samples from the two groups**

**Mean *m/z *value**	***p*-value**	Tendency*
3163.4	0.008	↓
1454.2	0.009	↑
3154.4	0.012	↓
2621.9	0.013	↓
5378.5	0.023	↓
1062.1	0.035	↑
3016.1	0.042	↓
5435.2	0.042	↓
2213.2	0.045	↓

*Tendency, *m/z* value intensity trend between the two groups: ↑, higher intensity in group B than A; ↓, lower intensity in group B than A. *m/z*, mass-to-charge ratio. A, the orthodontic patients without periodontitis; B, the orthodontic patients with periodontitis.

**Figure 4:**
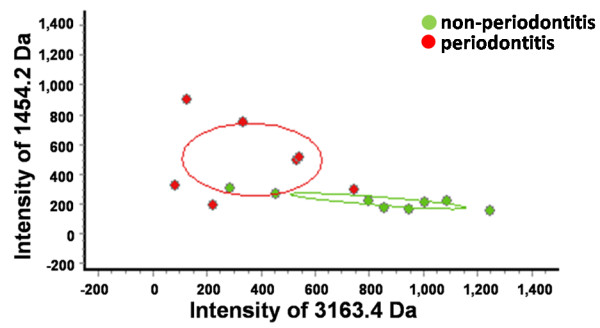
**Scatter plots of the two groups generated by combining the 3163.4-Da and 1454.2-Da proteins.** The scatter plots showed a well-fitting curve.

The most significant differences were exhibited by the 3163.4- and 1454.2-Da proteins (*p* <0.01); the fit of the other combinations was not as good. Thus, we chose these two peptides to establish a fitted curve between the two groups (Figure 4). Samples from the two groups were well-separated, indicating that the fitting results were satisfactory.

Moreover, using LTQ-Orbitrap-MS detection, eight (1062.1, 1454.2, 2213.2, 2621.9, 3016.1, 3154.4, 3163.4, 5378.5, and 5435.2 Da) of the nine differentially-expressed peptides were successfully identified (Table 2).

**Table 2:** Sequences of the differentially-expressed peptides

**Mean *m/z *value**	**Peptide name**	**Peptide sequence**
3163.4	F2 Prothrombin precursor	LRPLFEKKSLEDKTERELLESYIDGR
1454.2	SERPINA1(PRO2275)	PLFMGKVVNPTQK
3154.4	FGA Isoform 1 of fibrinogen alpha chain precursor	LIGQIVSSITASLR
2621.9	FGA Isoform 1 of fibrinogen alpha chain precursor	SYKM*ADEAGSEADHEGTHSTKRGHAKSRP
5378.5	VWCE Isoform 1 of von Willebrand factor C and EGF domain-containing protein precursor	APVNCSSCPGPPTASPSRPVLHLLQLLLRTNLM*KTQTLPTSPAGAHGPHSLA
1062.1	ITIH4 Isoform 2 of inter-alpha-trypsin inhibitor heavy chain H4 precursor	SEMVVAGKLQ
3016.1	280-kDa protein	LGVSPPPGAVLVLHSLPLEFPLAM*AFAEQ
5435.2	Unknown peptide-identification failure	
2213.2	ACTB Actin, cytoplasmic 1	DLYANTVLSGGTTMYPGIADR

*m/z*, mass-to-charge ratio.

The English in this document has been checked by at least two professional editors, both native speakers of English. For a certificate, please see: http://www.textcheck.com/certificate/OlkIGD.

## Discussion

Adult patients with periodontal disease exhibit tooth malocclusions such as flaring of the anterior segment. This unaesthetic appearance and/or dysfunction are the primary reasons for undergoing periodontal-orthodontic treatment using a multidisciplinary approach [24]. Combined orthodontic and periodontal treatment does not compromise the therapeutic effectiveness of the latter [25]. Indeed, this combination may be beneficial since it leads to increased bone resorption stability, a lack of incisor flaring, and improvements in bone defects [26]. However, some authors argue that orthodontic treatment is unsuitable in chronic periodontal patients because bacterial plaque is an etiologic factor in the development of periodontitis and the presence of orthodontic appliances facilitates plaque growth and maturation. Thus, the periodontal condition could worsen after orthodontic treatment [27]. Therefore, we examined periodontal-orthodontic patients with a view to elucidating the differences between them and non-periodontal orthodontic patients.

Such differences could be caused by inflammatory factors related to periodontitis or other protein factors [28], which may represent candidate biomarkers. A candidate biomarker is defined as an informative signal associated with a specific condition. The specificity and sensitivity of a biomarker describe its usefulness in diagnosing a specific condition or predicting its progress [29]. An effective biomarker should be measurable in an accessible body fluid, such as serum, urine, or saliva [30]. Saliva contains abundant proteins, peptides, and other small molecules [8]. Thus, the salivary peptide spectrum may be applied widely in the diagnosis and monitoring of oral diseases. Progress has been made in screening for not only oral diseases such as oral cancer [17], but also systematic conditions such as gastric cancer [31] and breast cancer [13]. Studies of periodontitis using proteomics [32,33] have been performed, and the demand for periodontal-orthodontic combination treatment is increasing. However, little research on the salivary peptide spectrum in the periodontal-orthodontic area has been conducted.

In the present study, we used MALDI-TOF MS-based proteomic methods and WCX magnetic beads to examine all 24 saliva samples. Nine peaks that differed significantly were found (Figure 2), of which two (3163.4 and 1454.2 Da) exhibited the most significant differences (*p* <0.01). Orthodontic treatment is a prolonged procedure. And in our previous study, alterations were found in salivary proteins due to different orthodontic treatment durations [34]. In this study, the peak intensities of the three different groups showed differential expression of salivary peptides, indicating the orthodontic treatment could contribute to the change of salivary peptidome. Noticeably, significant differences were investigated between the two orthodontic groups. This suggested that differential expression of salivary peptidome profile did exist between orthodontic patients with and without periodontitis. Thus, this method provides a new tool for analyzing the effect of periodontitis on orthodontic treatment.

The peptide sequence identifications made in this study have led to interesting speculations. The 3163.4-Da peptide was identified as F2 prothrombin precursor. Thrombin is a ‘trypsin-like’ serine protease protein encoded by the *F2* gene. Beyond its key role in the dynamic process of thrombus formation, thrombin has the potential to exert actions such as inflammation and leukocyte recruitment [35]. Moreover, isoform 1 of fibrinogen alpha chain precursor (FGA), which was the predicted identity of the 3154.4- and 2621.9-Da peptides, is encoded by *FGA*. It is cleaved by thrombin to form fibrin, indicating that it interacts with thrombin. Some studies [36] suggested epithelial thrombomodulin (TM), which binds to thrombin and converts it from a procoagulant protease to an anticoagulant enzyme, increased in gingival crevicular fluid of individuals with chronic periodontitis. Moreover, gingipains, the major periodontopathic bacterium Porphyromonas gingivalis-derived cysteine proteases, lead to the degradation of endothelial TM. In addition, reduced expression of TM was found in gingival microvascular endothelia in patients with periodontitis [37], and this may be involved in the pathogenesis of periodontitis. Thus, these differential expression patterns of altered proteins may have originated from periodontitis-associated inflammation or differences in bone metabolism between orthodontic patients with and without periodontitis. Ultimately, our aim is to determine the protein or gene from which a peptide is derived; however, this is complex. When the distribution of the peptides with low molecular weight were intended to match to the mass spectrometry spectra relative peak area, it should be noted that these peptides had complicated origins. Peptides in saliva could be secreted peptides and proteolytic fragments of related proteins [38]. Moreover, these components are subject to secondary modifications from distinctive protein families. Thus, a peptide sequence usually does not exclusively define a single protein.

As these specific peptides were investigated using this relatively new combined method with no validation using other techniques, thus, a larger sample size and repeated trial or trial using other techniques are needed to confirm the significant differences in peptide mass peaks found in this study and its reproducibility. The establishment of a relatively complete protein-peptide spectrum database will facilitate the determination of both the source of the salivary protein profile variation and the mechanism thereof. Moreover, the analysis of saliva is inherently challenging because of the wide protein concentration range therein [29] and the presence of multiple post-translational modifications [39]. Further research into the composition of saliva will likely provide novel tools for investigations of physiological and pathophysiological states.

## Conclusions

In conclusion, our data suggest that the salivary protein profiles of periodontal, non-periodontal orthodontic patients and periodontitis patients exhibit differential mass spectra peak intensities (i.e., the peptide profile is altered in cases of periodontitis). This represents a new method of analyzing the effect of periodontitis on orthodontics and orthodontic contribution to the alterations of salivary peptidome profile, ultimately leading to increased treatment efficacy. However, expansion of the orthodontic patient dataset and the identification of additional candidate biomarkers are necessary to establish an effective diagnosis and monitoring model for periodontal-orthodontic treatment.

## Methods

### Ethics statement

This study was approved by the Peking University Biomedical Ethics Committee. Adult subjects and parents of pediatric subjects signed an informed consent form before participating in the study.

### Subjects

Patients seeking treatment at the Stomatology School of Peking University were recruited in November, 2011. All 24 study subjects were generally ensured systemically healthy via testing of serum basic biochemical items and inquiring medical history, and those who presented with caries, diseases of the oral mucosa, or oral cancers were excluded. Recorded clinical periodontal parameters of the subjects included bleeding on probing (BOP),probing pocket depth (PPD) and clinical attachment level (CAL). Chronic periodontitis was diagnosed in patients who had six sites BOP on different teeth with PPD≥6 mm and CAL>5 mm. Control orthodontic patients had <10% of sites BOP and PPD<3 mm. And all the periodontitis patients manifested to be moderate degree of chronic periodontitis. Then the patients were divided into three groups according; that is, periodontal-orthodontic, non-periodontal orthodontic groups and patients with periodontitis but no orthodontic treatment. The characteristics of the subjects are presented in Table 3.

**Table 3:** Demographic information for the two groups

**Group**	**Sample size**	** Age**	
		**Mean**	**SD**
orthodontic patients without periodontitis	8	35.4	1.3165
orthodontic patients with periodontitis	8	33	2.2509
patients with periodontitis	8	34.55	2.2181

The treatment procedure for all subjects involved the use of a fixed appliance, followed by alignment, leveling, and refinement procedures. All patients were asked to maintain good oral hygiene during treatment and were counseled regarding oral hygiene and tooth brushing both before treatment and at each visit.

### Saliva collection and processing

All individuals were asked to rest for 15 min before saliva collection at 8:30 am, and not to eat or drink after dinner the previous evening or to brush their teeth on the collection day morning. The subjects sat upright in a quiet room and were required to put the tip of their tongue against the sublingual caruncle without straining. Thus, the saliva, which was received in a paper cup for the first 5 min, could run from the mouth, and we collected 6 mL of the spontaneous saliva flow in a 50-mL centrifuge tube. During the collection procedure, patients were asked not to speak. Immediately after collection, the unstimulated whole saliva samples were kept on ice and then centrifuged at 9000 × *g* for 7 min at 4°C to remove insoluble materials, cells, and debris. Ethylene diamine tetraacetic acid (1 mM; Sigma, St. Louis, MO, USA) and 1 mM phenylmethyl sulfonylfluoride (Sigma) were added to the sample supernatants to inhibit protease activity. Protein concentrations were determined using the Lowry assay and ELx808 Protein Assay (BioTek, Hercules, CA, USA). The supernatants were kept at −80°C for further analysis.

### Reagents and instruments

The WCX magnetic bead kit (SPE-C; Bioyong Tech, Beijing, China), alpha-cyano-4-hydroxycinnamic acid (HCCA), MALDI-TOF MS (Bruker Bio-sciences, Bremen, Germany), 100% ethanol (chromatographic grade), and 100% acetone (chromatographic grade) were freshly prepared.

### WCX fractionation and MALDI-TOF MS

The low-molecular-weight (LMW) salivary peptidome contains enormous and important biological information and they should be a rich source of specific candidate biomarkers [13]. Unlike 2D-PAGE analysis, which only allows the separation of proteins with molecular weight in a range of 20–300 kDa, the magnetic bead-based profiling MS technologies could cover a range of 1–250 kDa [29]. Moreover, the high-throughput nature of MALDI-TOF MS makes fast screening for novel candidate biomarkers possible, and this method is cost effective and can be easily adopted [31]. Besides, this combined method only required 20ul processed sample for generating enough proteins peaks profiling by MALDI-TOF-MS.

The WCX magnetic bead kit suspension was mixed by shaking. After eluting and beating, the magnetic beads were separated from the protein and the eluted peptide samples were transferred to a clean 0.5-mL sample tube for further analysis by MS.

Five microliters of HCCA substrate solution (0.4 g/L, dissolved in acetone and ethanol) and 0.8-1.2 μL of eluate were mixed. Next, 0.8-1.2 μL of this mixture was applied to a metal target plate and dried at room temperature. Finally, the prepared samples were analyzed by MALDI-TOF MS. Peptides with a molecular weight in the range of 1000–10000 Da were collected, and 400 shots of laser energy were used. Peptide mass fingerprints were obtained by accumulating 50 single MS signal scans.

### Identification of differentially expressed candidate biomarkers

The sequences of peptides expressed differentially between the two groups were determined by nano-liquid chromatography-electrospray ionization-tandem mass spectrometry (nano-LC/ESI-MS/MS) using a setup consisting of an Aquity UPLC system (Waters) and an LTQ Obitrap XL mass spectrometer (Thermo Fisher) equipped with a nano-ESI source. The obtained chromatograms were analyzed with BioworksBrowser 3.3.1 SP1, and the resulting mass lists were used in a database search with Sequest™ [IPI Human (3.45)]. The parameters used to generate the peak list were: parent ion and fragment mass relative accuracy (50 μg/g and 1 Da, respectively).

### Statistical analysis

A two-tailed *t*-test and Student’s *t*-test were used to compare peptide peak intensities between the two groups. Data were analyzed using the BioExplorer statistical package (BioyongTech). A *p*-value <0.05 was considered significant.

## Competing interests

The authors declare that they have no competing interests.

## Authors’ contributions

LJX and CF conceived of the idea for the peptidomic study and participated in its design. ZJN and ZSN carried out a major portion of the data analysis and drafted the manuscript. LRX, ZH and WXD carried out the sample collection and extraction. ZYH and DN carried out the data statistical analysis and a portion of the data analysis. CT provided the orthodontic patients. All authors read and approved the final manuscript.

## References

[B1] Kim YI Kim MJ Choi JI Park SB A multidisciplinary approach for the management of pathologic tooth migration in a patient with moderately advanced periodontal disease Int J Periodontics Restorative Dent 2012 32 225 230 22292151

[B2] Zetu I Ogodescu E Zetu L Stratul SI Rusu D Talpos S Ogodescu A [An interdisciplinary approach to complex orthodontic-periodontal clinical situations in adult patients] Rev Med Chir Soc Med Nat Iasi 2011 115 1262 1266 22276480

[B3] Tew JG El Shikh ME El Sayed RM Schenkein HA Dendritic cells, antibodies reactive with oxLDL, and inflammation J Dent Res 2012 91 8 16 10.1177/0022034511407338 21531918 PMC3232113

[B4] Dersot JM Gingival recession and adult orthodontics: a clinical evidence-based treatment proposal Int Orthod 2012 10 29 42 22257702 10.1016/j.ortho.2011.09.013

[B5] Beader N Ivic-Kardum M The role of cytomegalovirus infection in the pathogenesis of periodontal diseases Acta Clin Croat 2011 50 61 66 22034785

[B6] Tezal M Uribe S A lack of consensus in the measurement methods for and definition of periodontitis J Am Dent Assoc 2011 142 666 667 21628689 10.14219/jada.archive.2011.0250

[B7] Bader HI Salivary diagnostics in medicine and dentistry: a review Dent Today 2011 30 46 48 50–41; quiz 52–43. 21899018

[B8] Rosa N Correia MJ Arrais JP Lopes P Melo J Oliveira JL Barros M From the salivary proteome to the OralOme: comprehensive molecular oral biology Arch Oral Biol 2012 57 853 864 10.1016/j.archoralbio.2011.12.010 22284344

[B9] Punyadeera C Dimeski G Kostner K Beyerlein P Cooper-White J One-step homogeneous C-reactive protein assay for saliva J Immunol Methods 2011 371 19 25 21821037 10.1016/j.jim.2011.07.013

[B10] Punyadeera C Human saliva as a tool to investigate intimate partner violence Brain Behav Immun 2012 26 541 542 10.1016/j.bbi.2012.02.006 22388099

[B11] Li Y Denny P Ho CM Montemagno C Shi W Qi F Wu B Wolinsky L Wong DT The Oral Fluid MEMS/NEMS Chip (OFMNC): diagnostic and translational applications Adv Dent Res 2005 18 3 5 10.1177/154407370501800102 16000263

[B12] Schulz BL Cooper-White J Punyadeera C Saliva proteome research: current status and future outlook Crit Rev Biotechnol 2012 [Epub ahead of print]. 10.3109/07388551.2012.687361 22612344

[B13] Zhang L Xiao H Karlan S Zhou H Gross J Elashoff D Akin D Yan X Chia D Karlan B Wong DT Discovery and preclinical validation of salivary transcriptomic and proteomic biomarkers for the non-invasive detection of breast cancer PLoS One 2010 5 e15573 10.1371/journal.pone.0015573 21217834 PMC3013113

[B14] Ferraccioli G De Santis M Peluso G Inzitari R Fanali C Bosello SL Iavarone F Castagnola M Proteomic approaches to Sjogren's syndrome: a clue to interpret the pathophysiology and organ involvement of the disease Autoimmun Rev 2010 9 622 626 10.1016/j.autrev.2010.05.010 20462525

[B15] Giusti L Baldini C Ciregia F Giannaccini G Giacomelli C De Feo F Delle Sedie A Riente L Lucacchini A Bazzichi L Bombardieri S Is GRP78/BiP a potential salivary biomarker in patients with rheumatoid arthritis? Proteomics Clin Appl 2010 4 315 324 10.1002/prca.200900082 21137052

[B16] Wu JY Yi C Chung HR Wang DJ Chang WC Lee SY Lin CT Yang YC Yang WC Potential biomarkers in saliva for oral squamous cell carcinoma Oral Oncol 2010 46 226 231 10.1016/j.oraloncology.2010.01.007 20138569

[B17] Hu S Arellano M Boontheung P Wang J Zhou H Jiang J Elashoff D Wei R Loo JA Wong DT Salivary proteomics for oral cancer biomarker discovery Clin Cancer Res 2008 14 6246 6252 10.1158/1078-0432.CCR-07-5037 18829504 PMC2877125

[B18] Vitorino R de Morais Guedes S Ferreira R Lobo MJ Duarte J Ferrer-Correia AJ Tomer KB Domingues PM Amado FM Two-dimensional electrophoresis study of in vitro pellicle formation and dental caries susceptibility Eur J Oral Sci 2006 114 147 153 16630307 10.1111/j.1600-0722.2006.00328.x

[B19] Szabo GT Tihanyi R Csulak F Jambor E Bona A Szabo G Mark L Comparative Salivary Proteomics of Cleft Palate Patients Cleft Palate Craniofac J 2011 48 708 716 10.1597/09-161 21504360

[B20] Goncalves Lda R Soares MR Nogueira FC Garcia C Camisasca DR Domont G Feitosa AC Pereira Dde A Zingali RB Alves G Comparative proteomic analysis of whole saliva from chronic periodontitis patients J Proteomics 2010 73 1334 1341 10.1016/j.jprot.2010.02.018 20215060

[B21] Al-Tarawneh SK Bencharit S Applications of Surface-Enhanced Laser Desorption/Ionization Time-Of-Flight (SELDI-TOF) Mass Spectrometry in Defining Salivary Proteomic Profiles Open Dent J 2009 3 74 79 10.2174/1874210600903010074 19543543 PMC2697056

[B22] Schaub NP Jones KJ Nyalwidhe JO Cazares LH Karbassi ID Semmes OJ Feliberti EC Perry RR Drake RR Serum proteomic biomarker discovery reflective of stage and obesity in breast cancer patients J Am Coll Surg 2009 208 970 978 discussion 978–980. 10.1016/j.jamcollsurg.2008.12.024 19476873

[B23] Schwamborn K Krieg RC Grosse J Reulen N Weiskirchen R Knuechel R Jakse G Henkel C Serum proteomic profiling in patients with bladder cancer Eur Urol 2009 56 989 996 10.1016/j.eururo.2009.02.031 19282097

[B24] Derton N Derton R Perini A Gracco A Fornaciari PA Orthodontic treatment in periodontal patients: a case report with 7 years follow-up Int Orthod 2011 9 92 109 21419740 10.1016/j.ortho.2010.12.017

[B25] Lee JW Lee SJ Lee CK Kim BO Orthodontic treatment for maxillary anterior pathologic tooth migration by periodontitis using clear aligner J Periodontal Implant Sci 2011 41 44 50 10.5051/jpis.2011.41.1.44 21394296 PMC3051056

[B26] Boyer S Fontanel F Danan M Olivier M Bouter D Brion M Severe periodontitis and orthodontics: evaluation of long-term results Int Orthod 2011 9 259 273 21855438 10.1016/j.ortho.2011.06.004

[B27] van Gastel J Teughels W Quirynen M Struyf S Van Damme J Coucke W Carels C Longitudinal changes in gingival crevicular fluid after placement of fixed orthodontic appliances Am J Orthod Dentofacial Orthop 2011 139 735 744 10.1016/j.ajodo.2009.10.043 21640879

[B28] Hans M Hans VM Toll-like receptors and their dual role in periodontitis: a review J Oral Sci 2011 53 263 271 10.2334/josnusd.53.263 21959652

[B29] Al-Tarawneh SK Border MB Dibble CF Bencharit S Defining salivary biomarkers using mass spectrometry-based proteomics: a systematic review OMICS 2011 15 353 361 10.1089/omi.2010.0134 21568728 PMC3125555

[B30] Yang J Song YC Dang CX Song TS Liu ZG Guo YM Li ZF Huang C Serum peptidome profiling in patients with gastric cancer Clin Exp Med 2011 12 2 79 87 21739109 10.1007/s10238-011-0149-2

[B31] Wu ZZ Wang JG Zhang XL Diagnostic model of saliva protein finger print analysis of patients with gastric cancer World J Gastroenterol 2009 15 865 870 10.3748/wjg.15.865 19230049 PMC2653388

[B32] Mizuno N Niitani M Shiba H Iwata T Hayashi I Kawaguchi H Kurihara H Proteome analysis of proteins related to aggressive periodontitis combined with neutrophil chemotaxis dysfunction J Clin Periodontol 2011 38 310 317 10.1111/j.1600-051X.2010.01693.x 21226751

[B33] Rangé H Léger T Huchon C Ciangura C Diallo D Poitou C Meilhac O Bouchard P Chaussain C Salivary proteome modifications associated with periodontitis in obese patients J Clin Periodontol 2012 39 799 806 10.1111/j.1600-051X.2012.01913.x 22780105

[B34] Zhang J Zhou S Zheng H Zhou Y Chen F Lin J Magnetic bead-based salivary peptidome profiling analysis during orthodontic treatment durations Biochem Biophys Res Commun 421 844 849 22554512 10.1016/j.bbrc.2012.04.100

[B35] O'Brien M The reciprocal relationship between inflammation and coagulation Top Companion Anim Med 2012 27 46 52 10.1053/j.tcam.2012.06.003 23031455

[B36] Matsuyama T Tokuda M Izumi Y Significance of thrombomodulin release from gingival epithelial cells in periodontitis patients J Periodontal Res 2008 43 379 385 10.1111/j.1600-0765.2007.01033.x 18942187

[B37] Inomata M Ishihara Y Matsuyama T Imamura T Maruyama I Noguchi T Matsushita K Degradation of vascular endothelial thrombomodulin by arginine- and lysine-specific cysteine proteases from Porphyromonas gingivalis J Periodontol 2009 80 1511 1517 10.1902/jop.2009.090114 19722803

[B38] Amado F Lobo MJ Domingues P Duarte JA Vitorino R Salivary peptidomics Expert Rev Proteomics 2010 7 709 721 10.1586/epr.10.48 20973643

[B39] Pfaffe T Cooper-White J Beyerlein P Kostner K Punyadeera C Diagnostic Potential of Saliva: Current State and Future Applications Clin Chem 2011 57 675 687 10.1373/clinchem.2010.153767 21383043

